# Outbreak of H3N2 Influenza at a US Military Base in Djibouti during the H1N1 Pandemic of 2009

**DOI:** 10.1371/journal.pone.0082089

**Published:** 2013-12-05

**Authors:** Michael T. Cosby, Guillermo Pimentel, Remington L. Nevin, Salwa Fouad Ahmed, John D. Klena, Ehab Amir, Mary Younan, Robert Browning, Peter J. Sebeny

**Affiliations:** 1 United States Naval Medical Research Unit No. 3 (NAMRU-3), Cairo, Egypt; 2 Naval Medical Research Center (NMRC) – Frederick, Fort Detrick, Maryland, United States of America; 3 Johns Hopkins Bloomberg School of Public Health, Baltimore, Maryland, United States of America; 4 Emerging Infectious Diseases Program, China Office, United States Centers for Disease Control and Prevention, Beijing, China; 5 Walter Reed National Military Medical Center, Bethesda, Maryland, United States of America; 6 Naval Medical Research Center (NMRC), Silver Spring, Maryland, United States of America; University of Melbourne, Australia

## Abstract

**Background:**

Influenza pandemics have significant operational impact on deployed military personnel working in areas throughout the world. The US Department of Defense global influenza-like illness (ILI) surveillance network serves an important role in establishing baseline trends and can be leveraged to respond to outbreaks of respiratory illness.

**Objective:**

We identified and characterized an operationally unique outbreak of H3N2 influenza at Camp Lemonnier, Djibouti occurring simultaneously with the H1N1 pandemic of 2009 [A(H1N1)pdm09].

**Methods:**

Enhanced surveillance for ILI was conducted at Camp Lemonnier in response to local reports of a possible outbreak during the A(H1N1)pdm09 pandemic. Samples were collected from consenting patients presenting with ILI (utilizing a modified case definition) and who completed a case report form. Samples were cultured and analyzed using standard real-time reverse transcriptase PCR (rt-RT-PCR) methodology and sequenced genetic material was phylogenetically compared to other published strains.

**Results:**

rt-RT-PCR and DNA sequencing revealed that 25 (78%) of the 32 clinical samples collected were seasonal H3N2 and only 2 (6%) were A(H1N1)pdm09 influenza. The highest incidence of H3N2 occurred during the month of May and 80% of these were active duty military personnel. Phylogenetic analysis revealed that sequenced H3N2 strains were genetically similar to 2009 strains from the United States of America, Australia, and South east Asia.

**Conclusions:**

This outbreak highlights challenges in the investigation of influenza among deployed military populations and corroborates the public health importance of maintaining surveillance systems for ILI that can be enhanced locally when needed.

## Introduction

Respiratory illnesses, including influenza, contribute to a large disease burden in military settings. Deployed military personnel are at an increased risk of acquiring respiratory diseases, which are a significant threat due to historically high attack rates, rapid onset and difficulty controlling transmission in austere and crowded environments [1]. Despite high vaccination rates, influenza outbreaks with significant operational impact do occur [2]. It has been shown that deployed military personnel played an important role in the global spread of disease during the influenza pandemic of 1918-1919 [3]. During the H1N1 influenza A pandemic of 2009 [A(H1N1)pdm09], outbreaks among military personnel were quickly identified [4-6], reflecting the rapid global spread of disease. This early identification may reflect the effects of enhanced surveillance among military populations [7].

 The United States Department of Defense (DoD) conducts surveillance for influenza-like illness (ILI) among US personnel through a network of military bases in the United States and worldwide. This networked surveillance system collects epidemiological data and clinical specimens from patients presenting for a variety of illnesses. Collected data and specimens are fed into a centralized reporting system constituting part of a global surveillance network shared with the US Centers for Disease Control and Prevention (CDC). It is through this surveillance network that clusters of respiratory illness in various settings are detected, and a global snapshot of influenza trends, particularly regarding circulating and emerging strains, is developed [8,9]. This DoD surveillance system can be leveraged to investigate and respond to potential outbreaks at non-network military sites.

In this article we describe an operationally unique outbreak of H3N2 influenza identified during the A(H1N1)pdm09 pandemic at a remote, non-networked US military base in East Africa, detail the genetic characterization of the influenza virus strains isolated, and highlight some of the unique aspects of influenza surveillance in deployed military settings. 

## Materials and Methods

### Setting

Camp Lemonnier is a US military multiservice base in Djibouti which currently functions as the only fixed US base on the African continent. US military members live primarily on base (with some off-base privileges) and non-Djiboutian contractors also frequently live on base or in the local community. Several hundred locally employed host nationals work within the base and typically commute from residences within the surrounding local community. There is a continuous rotation of approximately 25-75 service members arriving from or departing to the US every 2-3 weeks. The Camp serves a considerable area of operation where security and capacity-building activities are conducted in the Horn of Africa and other areas of East Africa. During the months of April and May 2009, coinciding with the early stages of the A(H1N1)pdm09 pandemic [10], there was an anecdotal increase in reporting of respiratory illness at Camp Lemonnier and concern was raised as to whether these cases represented the beginning of a local A(H1N1)pdm09 influenza outbreak. As a result of this increased rate of respiratory illness and a desire to identify the etiology and scope of illness, enhanced surveillance for ILI cases was performed at the Expeditionary Medical Facility (EMF) at Camp Lemonnier, the main clinic serving the approximately 2,000 US service members and eligible US contractors. At the request of EMF leadership, resources of the US Naval Medical Research Unit-3 (NAMRU-3) in Cairo, Egypt were leveraged to provide epidemiologic and laboratory support. 

### Patient enrollment and historic medical review

In response to reports of increased respiratory illness, surveillance for ILI was expanded by modifying the standard ILI definition of cough and/or sore throat with fever (>100.4 °F) to include complaints of subjective fever. Patients prospectively identified through enhanced surveillance completed a case report form on which demographic data, travel history, clinical symptoms, and other relevant information was collected. 

To characterize the baseline of ILI prior to the time of the pandemic using the modified ILI definition, and to accurately identify all possible ILI cases that may not have been prospectively identified during the period of the suspected outbreak, the electronic medical record system in the EMF was queried to identify encounters of patients evaluated for respiratory illnesses or viral syndromes beginning March 1, 2009. The narrative medical records of identified patients were reviewed to determine whether or not each met the criteria for the modified definition of ILI. Data were extracted and imported into a case report form consistent with forms utilized in the EMF clinic for enhanced surveillance. We extracted data for encounters through the end of August, 2009, the apparent endpoint of the outbreak, after which time passive surveillance for ILI was resumed using pre-existing criteria. 

### Sample collection and processing

Nasopharyngeal (NP) swabs or nasal wash samples were collected only from patients identified prospectively during enhanced surveillance. These were stored in viral transport media at -70°C until tested. Preliminary testing of samples was conducted at Camp Lemonnier by NAMRU-3 staff using a Ruggedized Advanced Pathogen Identification Device (RAPID) 9200 PCR platform (Idaho Technology Inc, Salt Lake City, UT, USA) with reagents and kits provided by NAMRU-3. RNA was extracted from clinical specimens using the Qiagen QIAmp Viral RNA Mini Kit protocol (Qiagen, Valencia, CA, USA), extracted RNA was tested by real-time reverse transcriptase polymerase chain reaction (rt-RT-PCR) using SuperScript® III One-Step RT-PCR System with Platinum®Taq reagents and primers and probes from the CDC Human Influenza Virus rt-RT-PCR Detection and Characterization Panel [11]. Clinical specimens were retested as described above and results were confirmed at NAMRU-3 in Cairo, Egypt. Clinical specimens were not obtained from patients identified retrospectively.

### Virus isolation in cell culture

Clinical specimens were inoculated onto a monolayer of Madin Darby Canine Kidney (MDCK) (ATCC CCL-34) cells following standard cell culture and virus isolation techniques. The specimens were treated briefly with 5mg/ml gentamicin and 1000 U/ml penicillin (GIBCO, Carlsbad, CA, USA) prior to inoculation. Culture tubes with 80-90% confluent MDCK monolayer were inoculated with 100µl of each sample in growth media (DMEM with 2mM glutamine, 0.2% bovine serum albumin, 100U/ml penicillin, 100µg/ml streptomycin, 50µg/ml gentamicin, 2.5µg/ml amphotericin B and 2µg/ml TPCK trypsin). The tubes were incubated at 37°C in 5% CO_2_ and checked daily for 10 days for cytopathic effects (CPE). All cultures were screened by hemagglutination and rt-RT-PCR to detect viral presence. The obtained isolates were extracted for RNA and aliquots were stored at -70°C for further analysis. 

### RNA extraction and PCR

Extraction of RNA from culture isolates was performed using the Qiagen QIAmp Viral RNA Mini Kit protocol (Qiagen) followed by cDNA synthesis. 4µl of RNA was used with the Uni 12 universal primer [12] to generate the cDNA. The hemagglutinin (HA) and neuraminidase (NA) genes were amplified from the synthesized cDNA using specific primers (Table 1) using the GeneAmp High Fidelity PCR system protocol (Applied Biosystems, Foster City, CA, USA). The HA and NA obtained PCR products were visualized and purified by electrophoresis through 1.5% agarose and the QIAquick Gel Extraction kit (Qiagen). Those HA and NA genes from clinical samples that did not result in virus isolation were amplified from the extracted RNA previously described according to the CDC Protocol for Amplification and Sequencing of Influenza Viruses with M13 labeled primers as outlined by Deyde, et al.[15].

**Table 1:** Primers used for identification and sequencing of influenza strains.

**Name**	**Sequence**	**Reference**
H3HAR-362	TAA GGG TAA CAG TTG CTG	CDC
H3HAF567-NIMR	CTGAACGTGACTATGCCAAACAAT	WHO Collaborating Centre [13]
H3HAR-792	CAG TAT GTC TCC CGG TTT	CDC
H3HAR-1184	ATG GCT GCT TGA GTG CTT	CDC
H3Forward	AAG CAT TCC YAA TGA CAA ACC	CDC Diagnostic Assay 2005 [14]
AH3 A (Inf A)	CAGATTGAAGTGACTAATGC	Stockton, 1998 [14]
N2-MD-F1	AGC AAA AGC AGG AGT GAA AAT G	NAMRU-3
N2-MD-F500	CAA ACA AGT GTG CAT AGC ATG G	NAMRU-3
N2-MD-F1000	GGA AAC CCA GGA GTG AAA GG	NAMRU-3
N2-MD-R1300	ATG GAA CAG GCT CAT GGC	NAMRU-3

### Sequencing and phylogenetic analysis

Cycle sequencing was performed using the BigDye Terminator v3.1 Sequencing Kit (Applied Biosystems) and HA and NA amplification primers as well as internal primers (Table 1) to cover the full length of the HA and NA genes. The products of the sequencing reaction were purified using the BigDye XTerminator Purification Kit and sequenced on an ABI 3130xl genetic analyzer (Applied Biosystems). Sequences were edited and analyzed using Sequencher V4.10.1 (Gene Codes, Ann Arbor, MI, USA). A nucleotide database search was performed using blastN (basic local alignment search tool, Nucleotide) in GenBank. Bioedit software [16] was used for aligning the selected sequences. Phylogenetic and molecular evolutionary analysis was performed on MEGA 4 software [17] using the neighbor-joining method [18]. A maximum-likelihood composite model for nucleotide substitution and bootstrap analysis was implemented with 1000 replicates for sequence comparison [19]. The nucleotide sequences of the HA and NA genes isolated from influenza A H3N2 samples from this outbreak were compared to those of other selected influenza A H3N2 sequences from 2008 and 2009, available on GenBank, in a tree rooted to the 2009 influenza vaccine strain (A/Brisbane/10/2007). 

### Ethics statement

Data and clinical specimens were obtained under military command approval as public health surveillance, with analysis performed consistent with Department of Defense Directives (DoDD) 6590.2 and DoDD 6490.02E and as non-research exempt from Institutional Review Board (IRB) requirements in accordance with US Code of Federal Regulations (CFR) section 38 CFR 16 (‘‘The Common Rule’’). Identiﬁed health data were securely transmitted, processed, and retained on data systems compliant with DoD information assurance policies.

## Results

**Figure 1:**
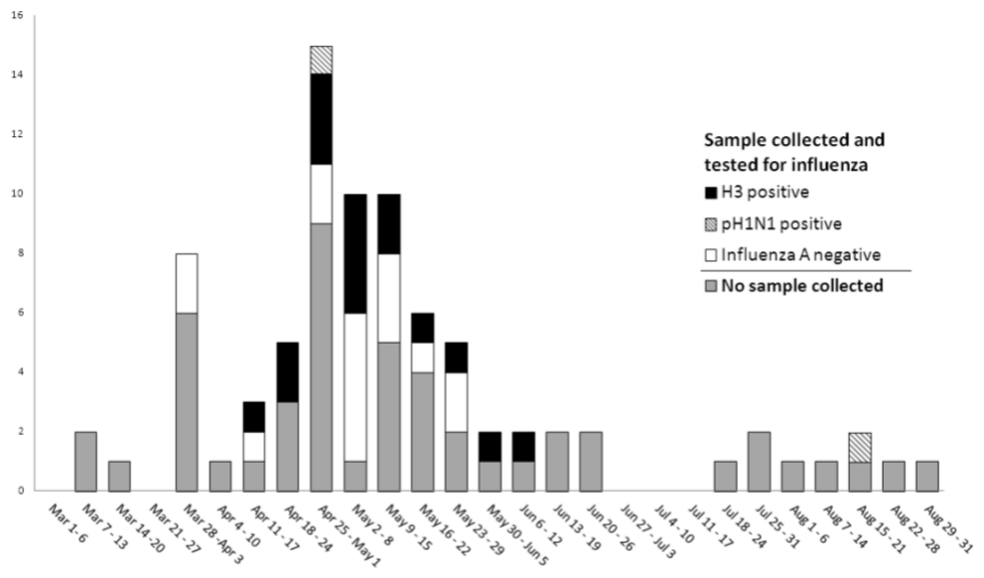
Incidence of ILI cases and confirmatory laboratory results by rt-RT-PCR, March 1- August 31, 2009.

From March 1 to August 31, 2009, 65 patients that met the modified ILI case definition were identified through enhanced surveillance (n=32) or through electronic medical record review (n=33). Overall, the median age was 32 years, and 57 (88%) were active duty personnel (Table 2). Preliminary field deployable PCR testing at the EMF revealed eight positive influenza A samples in specimens collected through May 8, 2009. Through the end of the surveillance period nasal washes or NP swabs were collected from a total of 32 patients and sent to NAMRU-3 for processing. Of the 32 samples collected, 25 (78%) were confirmed as seasonal H3N2 influenza by rt-RT-PCR and DNA sequencing. The majority of the H3N2 cases (n=20, 80%) were among active duty military with the remainder of cases from US contractors. The mean duration of symptoms prior to clinic visit was 2.5 days. Fourteen (56%) patients reported having been in close contact (within 6 feet) to a person with similar symptoms within the past 14 days and 8 (32%) patients reported having resided in a location outside Djibouti and the US for at least 24 hr during the deployment period. Figure 1 shows the frequency of ILI cases (meeting either standard or modified ILI definition) during the surveillance period. The initial H3N2 cases occurred in March and the highest incidence (based on an assumed stable base population) of laboratory-confirmed H3N2 seasonal influenza occurred during the month of May. Only two cases (6%) of confirmed A(H1N1)pdm09 (GenBank accessioning numbers: CY062432, CY062404) were identified throughout the surveillance period ending on August 31, 2009. All patients included in this study responded positively to conservative outpatient treatment and recovered without complications.

**Table 2:** Patient characteristics for influenza-like illness (ILI) and laboratory confirmed H3 cases.

	**ILI cases**		**Lab confirmed H3**	
**Patient Demographics, n (%)**	65	(100)	**25**	(100)
Age group, n (%)*				
18 - 25	14	(22)	3	(12)
26 - 37	24	(37)	14	(56)
38 and older	24	(37)	8	(32)
Median age (IQR)**	32	(27,41)	37	(32,43)
Male gender, n (%)	53	(82)	24	(96)
Active duty personnel, n (%)	57	(88)	20	(80)
Median time (days) from illness onset to clinic visit (IQR)	2	(1,3)	3	(1,4)
Travel history, n (%)†	7	(11)	8	(32)
Sick contacts, n (%)‡	7	(11)	6	(24)
**Clinical symptoms at presentation, n (%)**	65	(100)	25	(100)
Subjective fever	58	(89)	17	(68)
Documented fever§	18	(28)	8	(32)
Cough	51	(78)	20	(80)
Sore throat	32	(49)	7	(28)
Body aches	27	(42)	13	(52)
Rhinorrhea	20	(31)	6	(24)
Headache	19	(29)	10	(40)
Diarrhea and/or vomiting	6	(9)	3	(12)
Pneumonia	1	(2)	0	(0)

^*^ Three patients did not disclose age

^**^ Inter-quartile range

^†^ Defined as residing in a location outside Djibouti and the US for at least 24 hr during the deployment period

^‡^ Defined as close contact (within 6 feet) to a person with similar symptoms within the past 14 days

^§^ Defined as a clinically measured temperature >100.4°F

**Figure 2:**
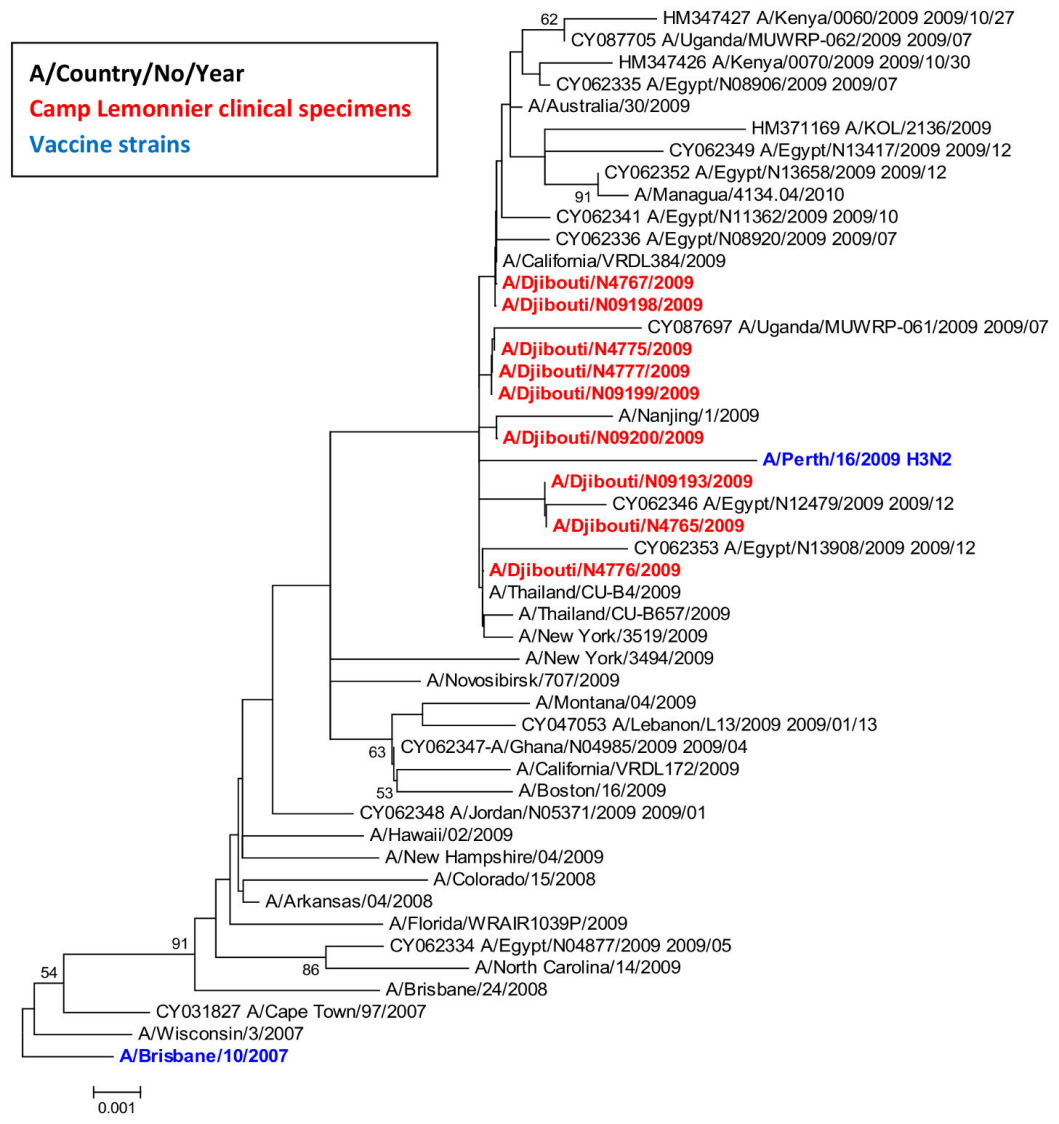
Phylogenetic analysis of H3N2 HA gene segments. *Phylogenetic tree of influenza A/H3N2 using the Neighbor-Joining method and Bootstrap test (1000 replicates). The evolutionary distances were computed using the Maximum Composite Likelihood method. The phylogenetic tree was generated using MEGA4 software. Phylogenetic analysis rooted to the 2009 influenza vaccine strain (A/Brisbane/10/2007).

**Figure 3:**
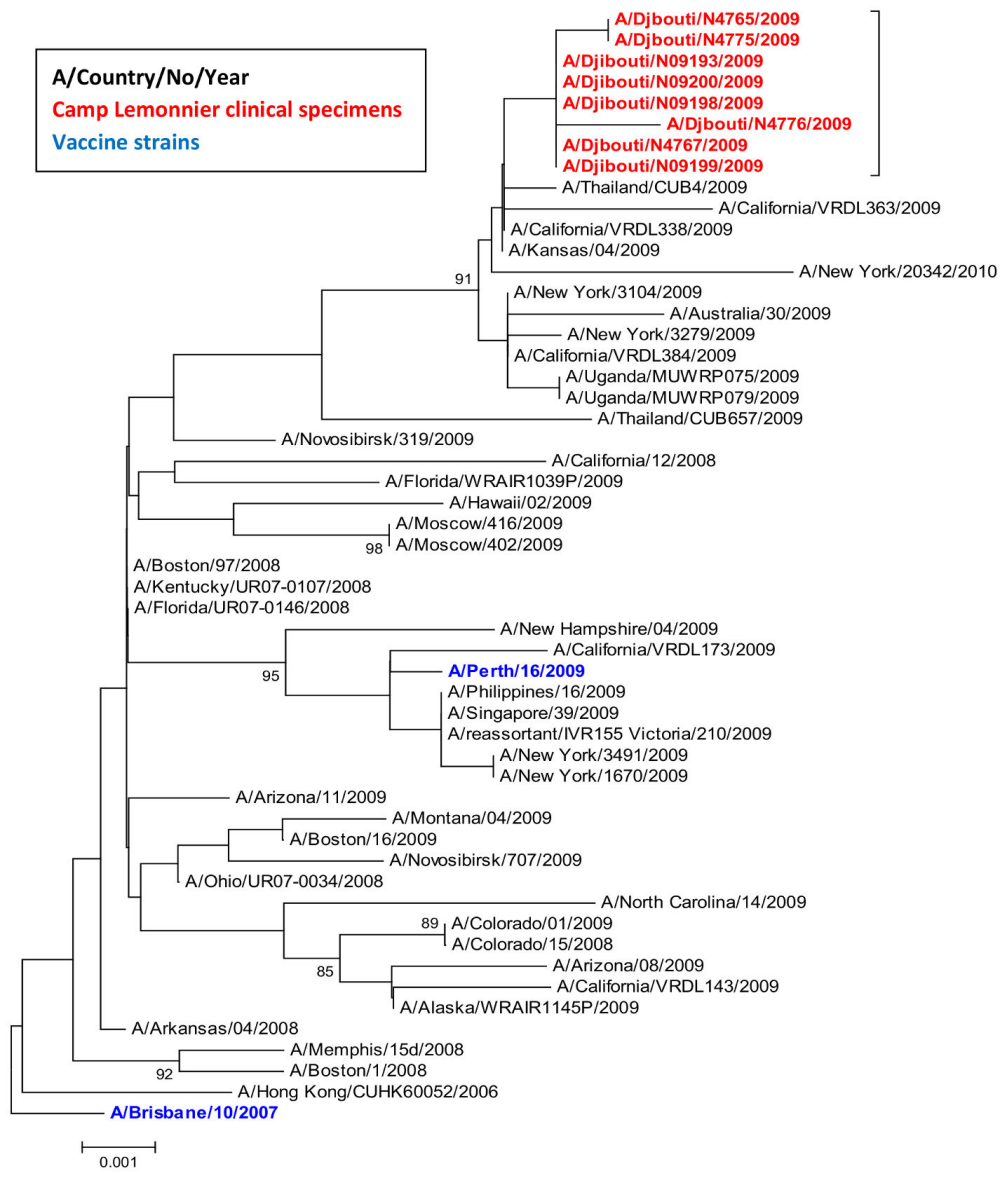
Phylogenetic analysis of H3N2 NA gene segments. *Phylogenetic tree of influenza A/H3N2 using the Neighbor-Joining method and Bootstrap test (1000 replicates). The evolutionary distances were computed using the Maximum Composite Likelihood method. The phylogenetic tree was generated using MEGA4 software. Phylogenetic analysis rooted to the 2009 influenza vaccine strain (A/Brisbane/10/2007).

We were able to obtain nine full-length HA gene sequences (GenBank accessioning numbers: : CY062329, CY062330, CY062331, CY062332, CY062333, KF626520, KF626521, KF626522, KF626523)and eight full-length NA gene sequences (GenBank accessioning numbers: KF626512, KF626513, KF626514, KF626515, KF626516, KF626517, KF626518, KF626519) from culture isolates of the 25 H3N2 positive clinical specimens (Figures 2 and 3). Comparison of the amino acid sequences of the HA gene from these isolates to the 2009 vaccine strain (A/Brisbane/10/2007) revealed seven amino acid differences: K158N, K173Q, V186G, N189K, N190D, T212A, and I362R. Phylogenetic reconstruction was performed using influenza sequences from this outbreak and H3N2 strains circulating worldwide between 2008 and 2009 (Figures 2 and 3). Analysis revealed that the HA gene sequences from the Camp Lemonnier isolates cluster together in a monophyletic group with genetic structures similar to 2009 strains from California, New York, Thailand, Australia, India, Nanjing, and Managua. They also cluster with strains from Uganda collected in July 2009 (CY087697 A/Uganda/MUXRP-061/2009 2009/07), and strains from Kenya and Egypt collected in October and December 2009 (HM 347426 A/Kenya/0070/2009 2009/10/30, CY062346 A/Egypt/N12479/2009 2009/12). These clusters were also not distantly separated from other influenza branches including the 2009 isolates from Novosibirsk, Ghana, and Lebanon. Analysis of the sequenced NA genes from the Djibouti influenza isolates revealed that none contained any of the established markers (E119V, R292K, and N294S) known to confer resistance to the neuraminidase inhibitors (NAIs) oseltamivir and zanamivir [20-22]. 

## Discussion

The ILI surveillance and outbreak investigation at Camp Lemonnier is of particular interest for several reasons. Given the timing of the outbreak with the emergence of A(H1N1)pdm09 influenza in North America in the Spring of 2009, and as Camp Lemonnier has a large flux of US military coming to and from the US, there were geopolitical concerns of introducing A(H1N1)pdm09 into areas where it had not yet been reported (and in areas where active screening of travelers was being conducted). Interest in preventing the potential spread of A(H1N1)pdm09 into the host nation of Djibouti as well as the uncertainty of its true clinical severity of illness led to heightened interest in identifying (and confirming) influenza cases, which in turn enhanced existing passive surveillance efforts. Such enhanced surveillance identified a cluster of seasonal H3N2 circulating in a predominantly US-based deployed population in Djibouti and identified the first reported lab-confirmed cases of A(H1N1)pdm09 in Djibouti. Success in identifying etiologic agents of this outbreak, initially believed to be a cluster outbreak of A(H1N1)pdm09, was in part due to the ability of staff at EMF to leverage and enhance a pre-existing passive surveillance system and to engage the support of a regional DoD reference laboratory (NAMRU-3) in outbreak response. On-site enhanced laboratory diagnostic capabilities would not only improve the sensitivity and specificity of surveillance systems in locations such as Djibouti but would also improve outbreak response and investigation.

It is important to note that 26% of the H3N2 cases identified in this cluster were among US contractor personnel who may live and interact within the local community and who are typically assigned to the base longer than active duty service members deployed for a period of only 6-12 months. Additionally, the fact that several of the patients infected with H3N2 (or A(H1N1)pdm09) influenza during the outbreak did not report travel history within seven days of symptom onset suggests that secondary transmission of both strains was occurring on the base. Secondary transmission on Camp Lemonnier is also supported by the sequenced HA genes from the H3N2 isolates obtained from the base having 100% identity to one another.

It is well established that influenza A (H3N2) viruses circulating between continents may seed epidemics in areas with established travel and trade infrastructure [23]. It is possible that the origin for the circulating viral strains in this epidemic may have been from cases with recent but unreported travel to areas of overlapping epidemics in South East Asia, a frequent destination of Camp Lemonnier active duty military staff and US contractors. Interestingly, unique characteristics of the influenza A H3N2 viruses circulating in 2008 and 2009, including mutations in the gene sequences coding neuraminidase proteins contributing to resistance to oseltamivir [24] and impacting the specificity of receptor binding [25] were not identified in our isolates.

It is also important to note that very limited data exist in regard to the seasonal aspect of influenza transmission in the Horn of Africa [26]. Global enhanced surveillance during the pandemic demonstrated considerable “off-season” transmission of influenza A viruses [27]. In other regions of the world, transmission of 2009 pandemic influenza has been shown to be similar to that of seasonal influenza in many aspects including similar sensitivities to temperature and humidity [28]. Co-circulation of A(H1N1)pdm09 and seasonal H3N2 influenza viruses led to mixed infections, with emergence of A(H1N1)pdm09 as the predominant strain, suggesting this viral strain has enhanced fitness [29]. In the outbreak we investigated, however, we did not identify any co-infections with A(H1N1)pdm09 or other seasonal influenza strains.

This article illustrates that deployed military personnel may serve as sentinel populations to identify the global spread or emergence of influenza strains. Due to crowding and other local geographical and operational conditions deployed personnel may be at higher risk for illness, and such military conditions may also create the potential for widespread transmission of influenza within the base population. As the US military population is often deployed to regions where little data exist on circulating influenza strains, surveillance can provide important surrogate data on circulating strains. Insights into influenza outbreak transmission dynamics from the global DoD influenza surveillance network (especially if local reporting can be performed in a timely manner) can be utilized at the local level as a framework for addressing emerging public health needs. In addition, as deployed US military members typically have vaccination rates exceeding 90%, ongoing surveillance among this population helps gauge vaccine effectiveness (and cross-protection) and may detect novel strains circulating in other regions of the world. Standardized reporting of surveillance data to the global DoD influenza surveillance network also ensures compliance with the International Health Regulations of 2005. This outbreak corroborates the public health importance of maintaining a simple, but flexible surveillance system for ILI in deployed US military populations. 
